# The Timing and Targeting of Treatment in Influenza Pandemics Influences the Emergence of Resistance in Structured Populations

**DOI:** 10.1371/journal.pcbi.1002912

**Published:** 2013-02-07

**Authors:** Benjamin M. Althouse, Oscar Patterson-Lomba, Georg M. Goerg, Laurent Hébert-Dufresne

**Affiliations:** 1 Department of Epidemiology, Johns Hopkins Bloomberg School of Public Health, Baltimore, Maryland, United States of America; 2 Mathematical, Computational, and Modeling Sciences Center, School of Human Evolution and Social Change, Arizona State University, Tempe, Arizona, United States of America; 3 Department of Statistics, Carnegie Mellon University, Pittsburgh, Pennsylvania, United States of America; 4 Département de Physique, de Génie Physique, et d'Optique, Université Laval, Québec, Québec, Canada; Pennsylvania State University, United States of America

## Abstract

Antiviral resistance in influenza is rampant and has the possibility of causing major morbidity and mortality. Previous models have identified treatment regimes to minimize total infections and keep resistance low. However, the bulk of these studies have ignored stochasticity and heterogeneous contact structures. Here we develop a network model of influenza transmission with treatment and resistance, and present both standard mean-field approximations as well as simulated dynamics. We find differences in the final epidemic sizes for identical transmission parameters (bistability) leading to different optimal treatment timing depending on the number initially infected. We also find, contrary to previous results, that treatment targeted by number of contacts per individual (node degree) gives rise to more resistance at lower levels of treatment than non-targeted treatment. Finally we highlight important differences between the two methods of analysis (mean-field versus stochastic simulations), and show where traditional mean-field approximations fail. Our results have important implications not only for the timing and distribution of influenza chemotherapy, but also for mathematical epidemiological modeling in general. Antiviral resistance in influenza may carry large consequences for pandemic mitigation efforts, and models ignoring contact heterogeneity and stochasticity may provide misleading policy recommendations.

## Author Summary

Resistance of influenza to common antiviral agents carries the possibility of causing large morbidity and mortality through failure of treatment and should be taken into account when planning public health interventions focused on stopping transmission. Here we present a mathematical model of influenza transmission which incorporates heterogeneous contact structure and stochastic transmission events. We find scenarios when treatment either induces large levels of resistance or no resistance at identical values of transmission rates depending on the number initially infected. We also find, contrary to previous results, that targeted treatment causes more resistance at lower treatment levels than non-targeted treatment. Our results have important implications for the timing and distribution of antivirals in epidemics and highlight important differences in how transmission is modeled and where assumptions made in previous models cause them to lead to erroneous conclusions.

## Introduction

The use of chemotherapy in the treatment of pathogenic disease places selective pressures on the pathogen to develop resistance to the treatment [1]. Since failure of chemotherapeutic agents in the treatment of influenza can cause large morbidity and mortality, much work has been done to understand the biology of – and assess the public policy regarding – resistance [2]–[5], this is especially important in the light of recent studies on the evolution of transmissibility of highly pathogenic avian influenza (H5N1) [6]–[9]. The most widely used antiviral agents, neuraminidase inhibitors (NIs) oseltamivir and zanamivir have demonstrated beneficial effects on pandemic and seasonal influenza strains, and thus play key roles in the planning of mitigation of epidemics [3], [5], [10]–[13]. Though fundamentally important to the transmission dynamics of infectious disease, the bulk of current studies examining the effects of treatment on resistance to therapies have ignored contact structure [14] and timing of treatment [15], [16]. Given the surprising and largely unpredictable evolutionary trajectories exhibited by influenza [6], the role of structure in populations may have significant effects on these trajectories. Here we employ network models of influenza transmission extending previous work [2] to incorporate the effects of contact structure and timing of antiviral treatment.

Network models are a robust framework for studying the transmission dynamics of infectious diseases in structured populations [17], [18]. Read & Keeling (2003) [14] examined the evolution of a pathogen on networks with varying contact structures, without the effects of treatment. They find differential levels of virulence depending on the clustering of the contact network. Previous studies have examined the role of treatments on networks of disease transmission. Pastor-Satorras (2002) [19] suggested targeting vaccination by node degree. While extremely effective in theory, identifying high degree individuals *a priori* is practically impossible. Cohen et al. (2003) [20] extended this idea to vaccinate an individual and one of the individual's contacts at random. Thus by design, the probability of identifying high degree individuals is greatly increased. This method has been shown empirically to be more effective at detecting influenza transmission early than by using a randomly selected group [21].

In addition to the problem of identifying individuals for efficient treatment, the timing of treatment plays directly into the evolution of resistance. Wu et al. (2009) [15] found that in a pandemic scenario with limited supplies of antivirals, it was beneficial to use a small amount of a secondary drug early in the epidemic to ‘hedge’ against the evolution of resistance. Hansen and Day (2011) [16] use optimal control theory to explore the effects of changing treatment over the course of an epidemic. They find that in a well-mixed, homogenous population it is optimal to fully treat a population as long as the timing is correct as they derive. While much important work has been done, the bulk of studies to this point have either ignored stochasticity [22], [23] or contact structure [14], [24] or both [25], the effects of which have been previously shown to be significant [26].

The goal of the present work is to combine network simulation models of evolution of pathogen resistance under chemotherapy and explore the effects of treatment timing and treatment regimes (targeted versus non-targeted) on the development and persistence of resistance. We focus on influenza and as we model resistance explicitly, we wish to answer three questions: one, to minimize resistance, should treatment be initiated at all in epidemics? two, if treatment is initiated, how does its timing affect the emergence and persistence of resistance in structured populations? and three, which treatment regime, targeted by degree or not, leads to the least amount of resistance? The approach taken here is novel in that our model combines stochasticity and population structure in assessing the role of treatment, and find results contrary to previous studies.

## Methods

### SIR Model Formulation

We extend an ordinary differential equation (ODE) model of treatment and resistance to influenza antivirals developed by Lipsitch et al. (2007) [2]. Whereas they considered both prophylactic and therapeutic treatment in well-mixed, homogenous populations, we consider only reactive treatment in structured populations. We limit our exploration to treatment because current guidelines suggest limiting prophylactic use of antivirals to individuals at high risk [5]. Our model features five possible states for individuals: susceptible (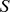), infectious and untreated (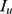), infectious and effectively treated (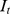), infectious with a resistant strain (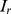), or recovered (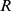). The dynamics then obey the following rules: susceptibles become infected at rates 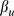, 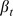, and 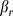 from untreated, treated and resistant individuals, respectively; wild-type infection (from 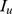 or 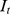 individuals) is treated with probability 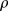; those treated develop *de novo* resistance with probability 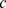; resistant infections (transmitted by 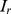) transmit only this strain (*i.e.*, no reverse mutation); and infectious individuals recover at rates 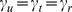, respectively. We assume treatment reduces transmissibility but does not affect the rate of recovery.

### Mean-Field Model

Disease propagation has been the subject of massive modeling efforts in recent network theory spanning multiple approaches and disease models [17], [27]–[29]. While the standard ODE treatment of epidemics is essentially a coarse-grained mean-field model of disease propagation in a population with homogeneous mixing, it has two main shortcomings in relation to realistic models of disease transmission: It neglects individual heterogeneity (*i.e.*, the variance of the node degree distribution 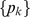) [27] as well as state correlations between neighboring nodes (*i.e.*, an infectious node is more likely to be connected to other infectious nodes) [30], [31].

To include individual heterogeneity we employ a network model of disease transmission. Here, in contrast to the standard 5-states modeled in the ODE system, one typically needs to introduce a higher-order compartmentalization where nodes are distinguished not only by their state, but also by their degree. Hence, instead of one equation for the fraction of susceptible individuals 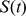 at time 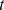, an infinite number of equations describes the fraction of susceptible nodes of degree 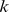, 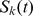, at time 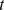. Correlations between nodes are then taken into account by coupling this system of equations to another system describing the evolution of the density of links stemming from susceptible nodes. To accurately reproduce features of real networks, we consider networks with heavy-tail degree distributions [32], [33]. Specifically, we use a binomial distribution leading into a power-law tail with exponential cut-off to avoid unrealistically high degree and infinite average excess degree (see Text S1). Such a heterogeneous distribution is more realistic in modeling influenza pandemics where there exists large variation in numbers of individual contacts across a population [34]. This is opposed to modeling outbreaks within small communities or schools, where there are natural lower and upper bounds to the numbers of possible contacts, not representing the variation seen across an entire population. Even so, in modeling transmission within small communities, it is still debated whether contact structure should feature heavy-tailed degree distributions [35]–[37] or not [38]; and, while several studies have indicated that networks with low coefficients of variation may be better for modeling influenza [38], others have not [34], [36]. Finally, heterogeneous distributions as employed here have been shown to influence the outcome of epidemics [27] and the efficiency of targeted treatment [19], [20]. The full mean-field model and ODE model equations and details of the degree distributions are given in .

### Including Stochasticity

Integrating the ODEs resulting from the mean-field analysis yields the possible final states of the dynamics. But such an analysis neglects the inherent stochastic nature of disease transmission. Standard epidemic models often only consider stochastic extinctions of a disease. When the contact structure of the population is known, the probability of extinction can be calculated [17]. However, in addition to stochastic extinction, our model dynamics also depend on the probability of treatment and mutation. Thus even though the mean-field model predicts a final state dominated by the resistant strain, a randomly picked trajectory will reach this state only if a mutation occurs (with probability 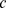), *i.e.*, infections must occur, then resistance is able to appear.

This becomes especially important if the resistant strain has a higher force of infection than the treated wild-type strain (*e.g.*, 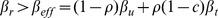) [39], [40]. In this case, even below the epidemic threshold of the treated wild-type strain, the development of resistance can occur and propagate. From the expected number of secondary infections caused by a quantity 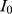 of initial infectious individuals and the total probability of transmission, 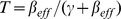 [17], one can calculate the probability, 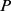, that an individual infected with a wild-type strain develops *de novo* resistance (details in Text S1):
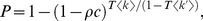
(1)where 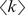 and 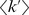 are the average degree and excess degree of the network, respectively [41]. Hence, Eq. (1) equals the probability of reaching a state where the resistant strain has emerged (assuming such a state is possible according to our mean-field analysis). Since the epidemic threshold is given by 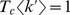, we set 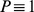 for 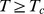. Note that Eq. (1) assumes that 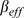 is such that 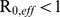, but 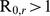. Finally, we note the generality of our model: parameter values chosen here are to illustrate and exaggerate the phenomena observed.

## Results

### Bifurcations and Treatment Timing

**Figure 1:**
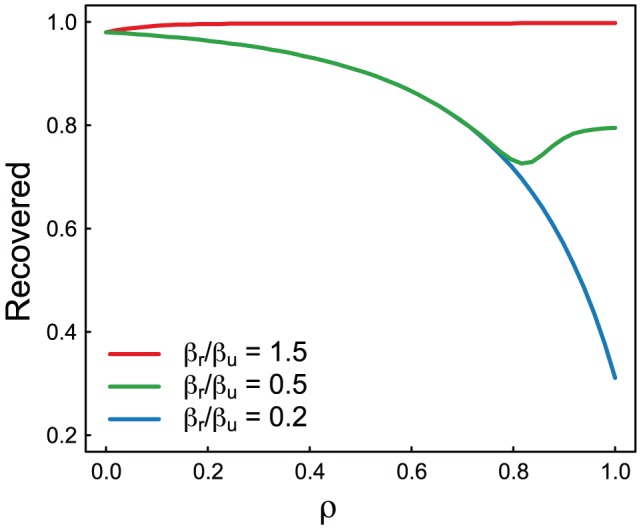
Final epidemic sizes depend on treatment levels and relative transmissibility. Figure shows the final epidemic size for various treatment levels when wild-type and resistant strains have differing transmissibilities. Treatment is only preferable when the wild-type strain is more transmissible than the resistant strain (*i.e.*: 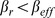). Model details given in [2]. **Parameters**: 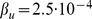, and 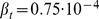, 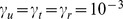, and 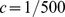.

We are interested in assessing the effects of the timing of antiviral treatment. If the resistant strain is less transmissible than the treated wild-type strain (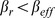), treatment will always be a good option and one must then concentrate on optimizing treatment efficiency (Figure 1). If the resistant strain is at least as transmissible as the treated wild-type strain (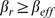), timing of treatment is crucial [15].

**Figure 2:**
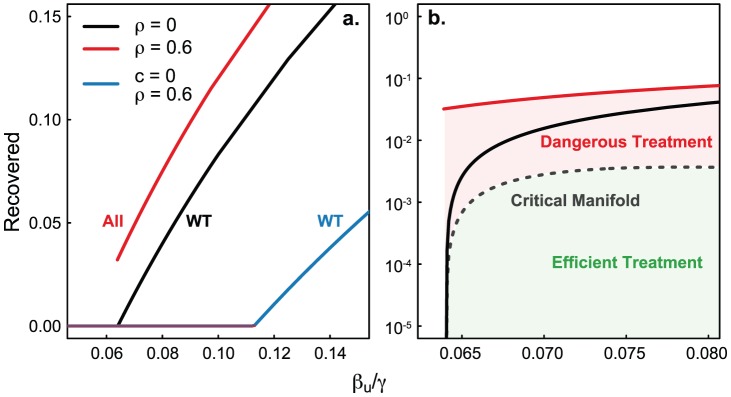
Final epidemic size and demonstration of the critical manifold. Results of the mean-field approximations. Panel **a** shows the final infected proportion as a function of 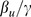 for all infections (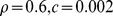) (red line), wild-type without treatment (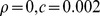) (black line) and wild-type without mutation (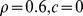) (blue line). Panel **b** demonstrates the critical manifold leading to dependence on initial conditions (dashed grey line). Treatment in the red region (“Dangerous Treatment”) results in emergence of resistance, while treatment in the green region (“Efficient Treatment”) can lead to eradication. Parameters: 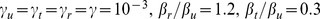.

Figure 2 shows the final epidemic size (proportion recovered) as a function of the untreated force of infection, 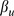, and corresponds to a situation when the resistant strain is more transmissible than the treated wild-type infections. For increasing values of 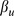 we see an expected increase in final epidemic size. However, the first bifurcation creates a regime of bistability where two final states can be reached for the same 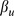 in stochastic simulations. Between the two possible branches, there exists a critical manifold corresponding to the curve of initial conditions (initial number infected, 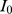) yielding equal expected epidemic sizes whether treatment is implemented or not (details in Text S1). Thus, depending on the number of infected individuals when treatment is initiated, we encounter one of three scenarios: one, where treatment is effective, *de novo* resistance is unlikely and there are few infections which eventually die out (this is the green area – “Efficient Treatment” – in Figure 2, panel **b**). In the second and third scenario (the red area – “Dangerous Treatment” – in Figure 2), treatment will most likely fail and result in either large incidence of resistant infections or a small outbreak of resistance in a depleted susceptible population (depending on the timing of this dangerous treatment). The derivation of the critical manifold is detailed in Text S1.

**Figure 3:**
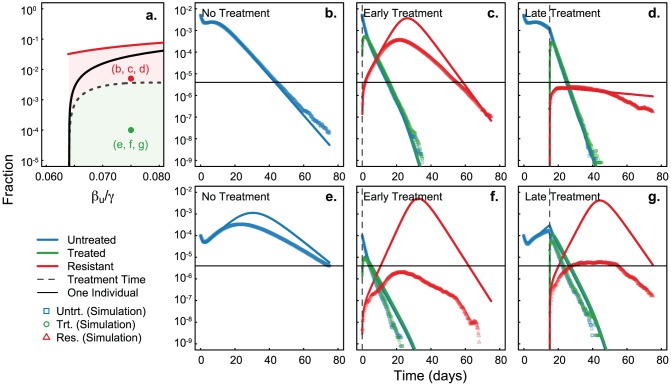
Treatment timing above and below critical manifold. Effects of treatment when initial conditions are above (panels **b, c, d**) and below (panels **e, f, g**) the critical manifold. Panel **a** is replicated from Figure 2, with each dot corresponding to the panels at right. Solid lines correspond to mean-field approximations, and points correspond to means of 100,000 simulations on networks of size 250,000. Horizontal black line corresponds to a mean of 1 infected individual in a network of 250,000 over 100,000 simulations. With no treatment the disease reaches a maximum and decays (panels **b** and **e**). Treatment is only effective early in the simulations when the initial conditions are under the critical manifold (panel **f** compared to panel **c**) as opposed to when the initial conditions are over the critical manifold (panel **g** compared to panel **d**). Parameters: 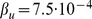, 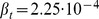, 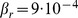, 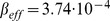, 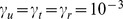, 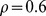, and 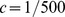.

Figure 3 demonstrates the behavior of the system in the regimes defined by this critical manifold. We see similar behavior for epidemics from both regimes when no treatment is applied (panels **b** and **e**). As observed in previous work [16], late treatment can be somewhat efficient if implemented after the peak of infections, such that the wild-type strain has depleted the pool of susceptibles to limit propagation of the resistant strain (panels **d** and **g**). However, since this implies that the bulk of the original epidemic has passed, this does not qualify as a truly efficient treatment regime. On the other hand, simulations (Figure 3, points) for early treatment of an epidemic with low initial number of infectious individuals appear significantly more efficient than predicted by the ODEs (Figure 3, solid lines, panels **c** and **f**).

This discrepancy is caused by the stochasticity of this system, or more precisely, by the mutation probability, 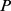. Such mathematical models based on mean-field approximations consider infinite populations in which a finite fraction of infectious individuals cause an infinite number of infections, resulting in an infinite number of treatments and an inevitable emergence of resistance. In finite populations, early treatment with low initial infections will cause only a small number of interventions resulting in a small probability of resistance emergence, 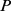. This is why the expected value of the prevalence of resistance is below one individual for all time in the simulations. Importantly, models without stochasticity would have not indicated treatment and failed to identify this efficient treatment regime (Figure 3). We note that presenting the per-epidemic average number of cases would have allowed the mean-field approximations to better align with simulations. This however would have ignored the role of stochastic extinctions including those due to successful treatment.

### Resistance and Targeted Therapy

Assuming treatment is expected to be efficient, we can explore two different forms of treatment: non-targeted, where 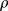 is a percentage of the population selected at random for treatment, and targeted, where 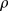 is a function of node degree (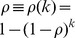), similar to Cohen et al. where an individual's probability of being treated depends on its degree [20].

**Figure 4:**
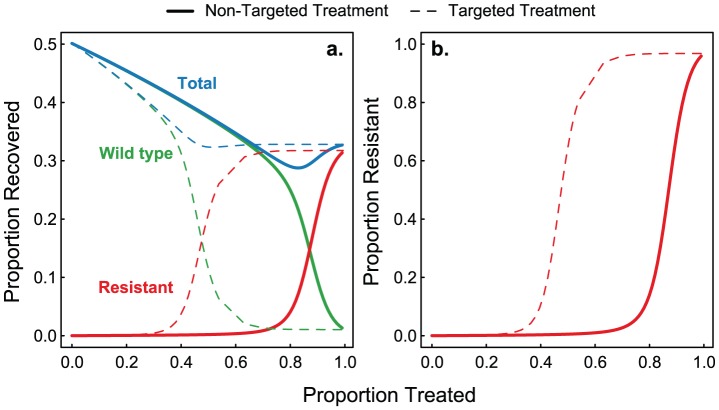
Comparison of random and targeted treatment. Panel **a** shows the final size for wild-type, resistant and both infections as a function of percentage treated, 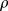, for targeted (dashed lines) and non-targeted (solid lines) treatment regimes. We see a transition from wild-type to resistant infections at a lower treatment percentage in the targeted treatment regime. Panel **b** shows the percent of total infection that is the resistant strain for the targeted (dashed line) and non-targeted (solid line) treatment. Parameters: 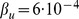, 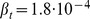, 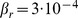, 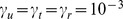, and 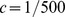.

We focus on scenarios where treatment would be indicated *a priori*; *i.e.*, when there is a fitness cost to resistance (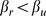). In the case when there is no cost of resistance (as explored above) treatment may or may not be optimal, however the results are qualitatively similar. Similar to previous studies [2], [4], we see a transition from wild type to resistant infections as treatment levels increase, and find a minimum in disease prevalence at intermediate levels of treatment. Interestingly, we see higher levels of resistance at lower treatment percentages in the targeted treatment regime. Figure 4 shows that under the non-targeted treatment regime, the resistant strain dominates when 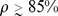, whereas under the targeted treatment regime, resistance is dominant when 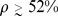. This happens because targeted treatment increases the chances of resistance occurring in high-degree nodes. Once resistant mutants arise in highly connected nodes, they will have a high probability of being widely transmitted. In addition to the take over of the resistant strain in the targeted treatment regime, we see high levels of total infection with increasing percentage treated due to treatment failure in the resistant cases.

Finally, we find the effects of treatment targeting to be robust to the network structure. Under a more homogenous degree distribution (binomially distributed) we find the difference between high- and low-degree individuals to be less than in the heterogeneous network, and thus targeting treatment by degree has a smaller effect. However, the results are qualitatively the same, with targeted treatment leading to higher levels of resistance at lower levels of treatment than non-targeted treatment (see Text S1). This finding is reassuring given the uncertainty in actual contact structures relevant to influenza transmission [34], [36], [38].

## Discussion

In the current study we wanted to answer three questions: one, to minimize resistance, should treatment be initiated at all in epidemics? two, if treatment is initiated, how does its timing affect the emergence and amount of resistance in structured populations? and three, which treatment regime, targeted by degree or not, leads to the least amount of resistance? We find potential bistability in the final epidemic size and deviations from mean-field approximations which would have misidentified optimal treatment timing. We find two scenarios: one, when the initial number infected is low (early in an epidemic), early treatment is preferable to late treatment, and two, when the initial number infected is high, treatment after the peak of epidemic is optimal to keep resistance low. Interestingly, this occurs at identical values of the force of infection (values of 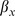), and indicates a strong dependence on initial conditions (number of cases at the onset of treatment) and thus on the timing of treatment. Given the uncertainty inherent in estimating epidemic prevalence, especially in emerging infections [42], caution must be taken when deciding to implement mass treatment.

In addition to the presence of this bifurcation and strong dependence on initial conditions we find large differences depending on the method used to allocate treatment. In accordance with previous results, we find a minimum in the total number of infections at intermediate levels of antiviral use. Surprisingly however, we find higher levels of resistance at lower levels of treatment in the targeted treatment case. This is due to the heterogeneity in contact structure wherein if those that are preferentially targeted for treatment (due to their high number of secondary contacts) develop *de novo* resistance, they have a large opportunity to spread the resistant strain. This is counter to previous results demonstrating that targeted treatment is optimal to keep absolute numbers of infecteds low. Thus, in structured populations, non-targeted treatment is preferable if resistance is to be minimized. This implies that in populations where the development of resistance is of concern, resources do not need to be spent on targeting treatment. We note two things: first, in cases where drugs are scarce, the amount of resistance expected to appear is low (Figure 4) and treatment targeted by node degree and factors not considered here (*i.e.*, treating teachers, healthcare workers, first-responders, etc.) is preferable to no treatment or non-targeted treatment. Second, non-targeted, or random treatment may be complicated by additional clinical factors also not considered here (*i.e.*, age, severity of illness, pregnancy, etc.); however, our results indicate that in cases where antivirals can be provided to a large fraction of the infected population, resource-intensive targeting by degree need not be employed and treatment should be initiated based on clinical factors alone.

The current work highlights the importance of including stochasticity and contact structure in epidemic models. Due to the bistability in final epidemic sizes, the mean-field approximation overestimated the number of resistant cases when treatment was initiated early and missed the efficient treatment when the initial numbers of infected are low. Additionally, we have shown that targeted treatment is not optimal due to the heterogeneous contact structure of the population. This is contrary to earlier studies demonstrating the efficiency of targeted treatment. While our results are qualitatively valid, and hold over multiple network types (see Text S1), more detailed models can and should be developed to study the effects of contact structure heterogeneity on the development of resistance. Parameters were chosen to be general, and give qualitative results, more accurate statistical estimation could be employed to improve the realism of the model.

The timing and targeting of antivirals for the treatment of influenza has important policy implications. Recent studies have demonstrated the facility with which highly pathogenic H5N1 can mutate to spread efficiently from human-to-human [6]–[9]. The development of resistance of H5N1 to common antiviral treatments, could have devastating consequences. We have demonstrated the danger of initiating treatment when the number of infected cases have surpassed a certain threshold (above and below the critical manifold), but have also demonstrated that spending resources on targeting treatment may not be necessary.

## Supporting Information

Text S1 **Supporting information.** Supporting Information includes: Model equations, analytical derivation of critical manifold, and additional parameter explorations.
(PDF)
