# Supplementary material for: The Timing and Targeting of Treatment in Influenza Pandemics Influences the Emergence of Resistance in Structured Populations
Source: PLoS Comput Biol. 2013 Feb 7;9(2):e1002912. doi: 10.1371/journal.pcbi.1002912 (PMC3567146; doi:10.1371/journal.pcbi.1002912)
Supplement: Text S1 — Supporting information. Supporting Information includes: Model equations, analytical derivation of critical manifold, and additional parameter explorations. (PDF) [file pcbi.1002912.s001.pdf]

Text S1: Supporting Information for The Timing and Targeting of Treatment in Influenza Pandemics Influences the Emergence of Resistance in Structured Populations

Benjamin M. Althouse, Oscar Patterson-Lomba, Georg M. Goerg, Laurent Hébert-Dufresne

ODE Model Equations

Our deterministic SIR model extends a framework presented by Lipsitch et al. (2008) [1]. We have five classes of individuals: susceptible (S), infectious and untreated (I_u), infectious and treated (I_t), infectious with a resistant strain (I_r) or recovered (R). Let γ_u, γ_t and γ_r be the recovery rate for untreated, treated and resistant infections, respectively and let c be the probability of a sensitive infection evolving *de novo* resistance to treatment. Let β_u, β_t, and β_r be the transmission parameters for untreated sensitive, treated sensitive, and resistant virus respectively. As in [1], treatment reduces the infectiousness but not the duration of infection. Let ρ be the fraction of drug-sensitive infections that receive treatment. The governing differential equations are then:

$$
\begin{aligned}
\dot{S} &= -(\beta_u I_u + \beta_t I_t)S - \beta_r I_r S \\
\dot{I}_u &= (\beta_u I_u + \beta_t I_t)(1-\rho)S - \gamma_u I_u \\
\dot{I}_t &= (\beta_u I_u + \beta_t I_t)\rho(1-c)S - \gamma_t I_t \\
\dot{I}_r &= (\beta_u I_u + \beta_t I_t)\rho c\, S + \beta_r I_r S - \gamma_r I_r \\
\dot{R} &= \gamma(I_u + I_t + I_r)
\end{aligned}
\tag{1}
$$

Mean Field Network Model Equations

To include individual heterogeneity we employ a network model of disease transmission. Here, in contrast to the ODEs in (1), one typically needs to introduce a higher-order compartmentalization where nodes are distinguished not only by their state, but also by their degree. Hence, instead of one equation for the fraction of susceptible individuals $S(t)$ at time t, we write an infinite number of equations for the fraction of susceptible nodes of degree k, $S_k(t)$, at time t. We obtain

$$\dot{S}_k(t) = -k\left(\beta_u\langle I_u\rangle + \beta_t\langle I_t\rangle + \beta_r\langle I_r\rangle\right) S_k(t) \tag{2}$$

where $\langle I_x\rangle$ is the probability, that a randomly chosen link among the k belonging to a susceptible node leads to an infectious individual of type $x \in \{$**u**ntreated, **t**reated, **r**esistant$\}$. Similarly,

$$\dot{I}_{u,k}(t) = k\left(\beta_u\langle I_u\rangle + \beta_t\langle I_t\rangle\right)(1-\rho)S_k(t) - \gamma_u I_{u,k}(t) \tag{3}$$

$$\dot{I}_{t,k}(t) = k\left(\beta_u\langle I_u\rangle + \beta_t\langle I_t\rangle\right)\rho(1-c)S_k(t) - \gamma_t I_{t,k}(t) \tag{4}$$

$$\dot{I}_{r,k}(t) = k\left(\beta_u\langle I_u\rangle + \beta_t\langle I_t\rangle\right)\rho c S_k(t) + k\beta_r\langle I_r\rangle S_k(t) - \gamma_r I_{r,k}(t) \tag{5}$$

$$\dot{R}(t) = \sum_k \gamma_u I_{u,k}(t) + \gamma_t I_{t,k}(t) + \gamma_r I_{r,k}(t)\ . \tag{6}$$

Our ability to evaluate this mean-field quantity directly depends on our ability to correctly follow the links stemming from susceptible nodes. To this end, we write a set of five ODEs for the density of the possible links (where a link between nodes of state X and Y is denoted as $[XY]$):

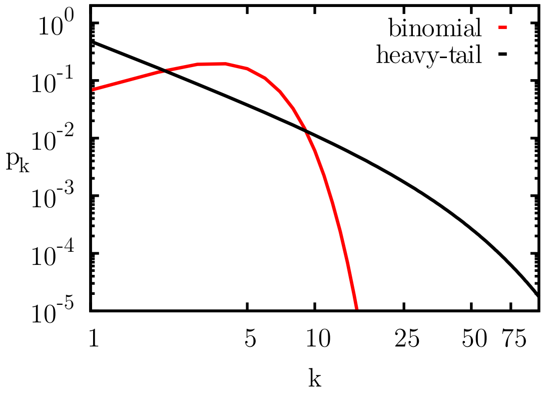

Supplemental Figure 1: **Degree distributions used in the main text.** The heavy-tail distribution (black line) corresponds to an initial binomial regime leading into a power-law tail with exponential cutoff (for mean degree $\langle k \rangle \sim 4.1$). The binomial network is given by the red line and has the same mean degree.

$$\dot{[SS]} = -2\left(\beta_u\langle I_u\rangle + \beta_t\langle I_t\rangle + \beta_r\langle I_r\rangle\right)\langle k'_s\rangle[SS] \tag{7}$$

$$\dot{[SI_u]} = -\left[\left(\beta_u\langle I_u\rangle+\beta_t\langle I_t\rangle+\beta_r\langle I_r\rangle\right)\langle k'_s\rangle+\beta_u+\gamma_u\right][SI_u] + 2\left(\beta_u\langle I_u\rangle+\beta_t\langle I_t\rangle\right)\langle k'_s\rangle(1-\rho)[SS] \tag{8}$$

$$\dot{[SI_t]} = -\left[\left(\beta_u\langle I_u\rangle+\beta_t\langle I_t\rangle+\beta_r\langle I_r\rangle\right)\langle k'_s\rangle+\beta_t+\gamma_t\right][SI_t] + 2\left(\beta_u\langle I_u\rangle+\beta_t\langle I_t\rangle\right)\langle k'_s\rangle\rho(1-c)[SS] \tag{9}$$

$$\dot{[SI_r]} = -\left[\left(\beta_u\langle I_u\rangle+\beta_t\langle I_t\rangle+\beta_r\langle I_r\rangle\right)\langle k'_s\rangle+\beta_r+\gamma_r\right][SI_r] + 2\left(\beta_u\langle I_u\rangle\rho c+\beta_t\langle I_t\rangle\rho c+\beta_r\langle I_r\rangle\right)\langle k'_s\rangle[SS] \tag{10}$$

$$\dot{[SR]} = -\left(\beta_u\langle I_u\rangle + \beta_t\langle I_t\rangle + \beta_r\langle I_r\rangle\right)\langle k'_s\rangle[SR] + \gamma_u[SI_u] + \gamma_t[SI_t] + \gamma_r[SI_r] \tag{11}$$

where $\langle k'_s\rangle$ is the average excess degree of susceptible nodes. Equations (2)–(11) are minimal to describe the system in the sense that they are sufficient to calculate all the mean-field quantities on which they depend. Through a simple averaging procedure, one obtains:

$$\langle k'_s\rangle = \frac{\sum_k k(k-1)S_k}{\sum_k kS_k}, \tag{12}$$

$$\langle I_x\rangle = \frac{[SI_x]}{2[SS]+[SI_u]+[SI_t]+[SI_r]+[SR]} \tag{13}$$

We evaluate (2)–(11) numerically to explore the transmission dynamics of this system.

Derivation of the Critical Manifold

If $\beta_r < \beta_{\text{eff}} = (1-\rho)\beta_u + \rho(1-c)\beta_t$, and treatment effects only transmissibility, we assume that it is always better to treat than not to treat. We also assume that we are not under the epidemic threshold of either the resistant or untreated strains, as the disease would then die out even without

treatment. In opposition, the case where $\beta_r > \beta_{\text{eff}}$, and both the resistant and untreated wild strains are above the threshold, can lead to an important dilemma: is treatment more likely to be effective at reducing total incidence or at creating resistance? This is problematic because the resistant strain has the opportunity to lead to a bigger epidemic than the untreated disease through treatment failure, so that treatment will actually have worsened the situation.

Here, we calculate the critical initial conditions of treatment $I(0)$ (i.e. the number of individuals in the I_u state at the beginning of treatment) for which treatment has approximately a 50/50 chance of being efficient or dangerous.

Above the epidemic threshold of the resistant strain, one individual infected with the resistant strain could spark an epidemic. Thus we simply calculate the probability of getting at least one mutated strain given that we begin treatment when $I(0)$ random individuals are currently infectious. We assume that we are below the epidemic threshold of the treated disease, as an epidemic of the resistant strain is always the more likely outcome.

Below this threshold, each of the $I(0)$ infectious creates a microscopic epidemic of size $1+\frac{T\langle k\rangle}{1-T\langle k'\rangle}$ [2], where $\langle k\rangle$ and $\langle k'\rangle$ are the average degree and excess degree of the network, respectively, and T is the total probability of transmission. Removing the original untreated infectious individuals leads to $I(0)\frac{T\langle k\rangle}{1-T\langle k'\rangle}$ new infections. From these, the probability of getting at least one mutation is directly obtained by

$$P_{muta}(I(0)) = 1 - (1-\rho\cdot c)^{I(0)\frac{T\langle k\rangle}{1-T\langle k'\rangle}}. \tag{14}$$

The critical initial conditions of treatment $I_c(0)$ is that $I(0)$, for which expected epidemic sizes are equal with and without treatment. The critical probability of mutation $P_c := P_{muta}(I_c(0))$ must thus satisfy

$$P_c R_r + (1-P_c)R_t = R_u. \tag{15}$$

where R_u, R_t and R_r are the expected epidemic size for the untreated, treated without mutation and treated with mutation cases, respectively. Solving for $I_c(0)$ gives

$$I_c(0) = \frac{1-T\langle k'\rangle}{T\langle k\rangle}\frac{\log\left(1-\frac{R_u-R_t}{R_r-R_t}\right)}{\log(1-\rho\cdot c)}. \tag{16}$$

Finally, note that the total probability of transmission T is easily approximated from the effective transmission rate of the treated disease, i.e. $\beta_{\text{eff}} = (1-\rho)\beta_u + \rho(1-c)\beta_t$,

$$T = \frac{\beta_{\text{eff}}}{\gamma+\beta_{\text{eff}}}. \tag{17}$$

Details of Network Simulations

To perform MC simulations of the model, we have generated networks of size N with the degree distribution $\{p_k\}$ in Figure 3 via the following numerical algorithm:

i. draw a sample $\{k_i\}$ of size N from distribution $\{p_k\}$ under the condition that $\sum_{i=1}^{N} k_i$ is even;

ii. for each i, produce k_i stubs tagged as i;

iii. randomly link all stubs in pairs (i,j), thus linking nodes i and j.

This is the so-called Configuration Model with allowed loops and self-links [3]. Each and every network generated by this procedure is accepted and kept in the results, as they are part of the

canonical ensemble considered by the mean-field approach of the formalism. For every generated network, a fraction $I(0)$ of individuals are randomly chosen to be initially infectious and the dynamics are then simulated in a discrete time propagation simulation valid for a time step $\Delta t \to 0$ (we choose Δt such that $\beta_x \Delta t$ and $\gamma_x \Delta t$ are less than 10^{-3} for all x):

i. at each Δt, every susceptible neighbor S of every infectious individual I_x is infected with probability $\beta_x \Delta t$;

ii. wild strain infections are treated with probability ρ leading to mutation with probability c;

iii. at each Δt every infectious individual I_x recovers with probability $\gamma_x \Delta t$.

Effects of Network Structure

The main results of the paper are robust to changes in network structure. Supplemental Figures 2 and 3 are analogous to Figures 3 and 4 in the main text but with binomial degree distributions (Supplemental Figure 1). Treatment when the system is above the critical manifold results in widespread resistance, while treatment below the critical manifold reduces epidemic size. When treatment is initiated early, resistance is minimized (Supplemental Figure 3).

References

1. Lipsitch M, Cohen T, Murray M, Levin BR (2007) Antiviral resistance and the control of pandemic influenza. PLoS Medicine 4.

2. Newman MEJ (2002) Spread of epidemic disease on networks. Phys Rev E 66: 016128.

3. Newman ME, Strogatz SH, Watts DJ (2001) Random graphs with arbitrary degree distributions and their applications. Phys Rev E 64: 026118.

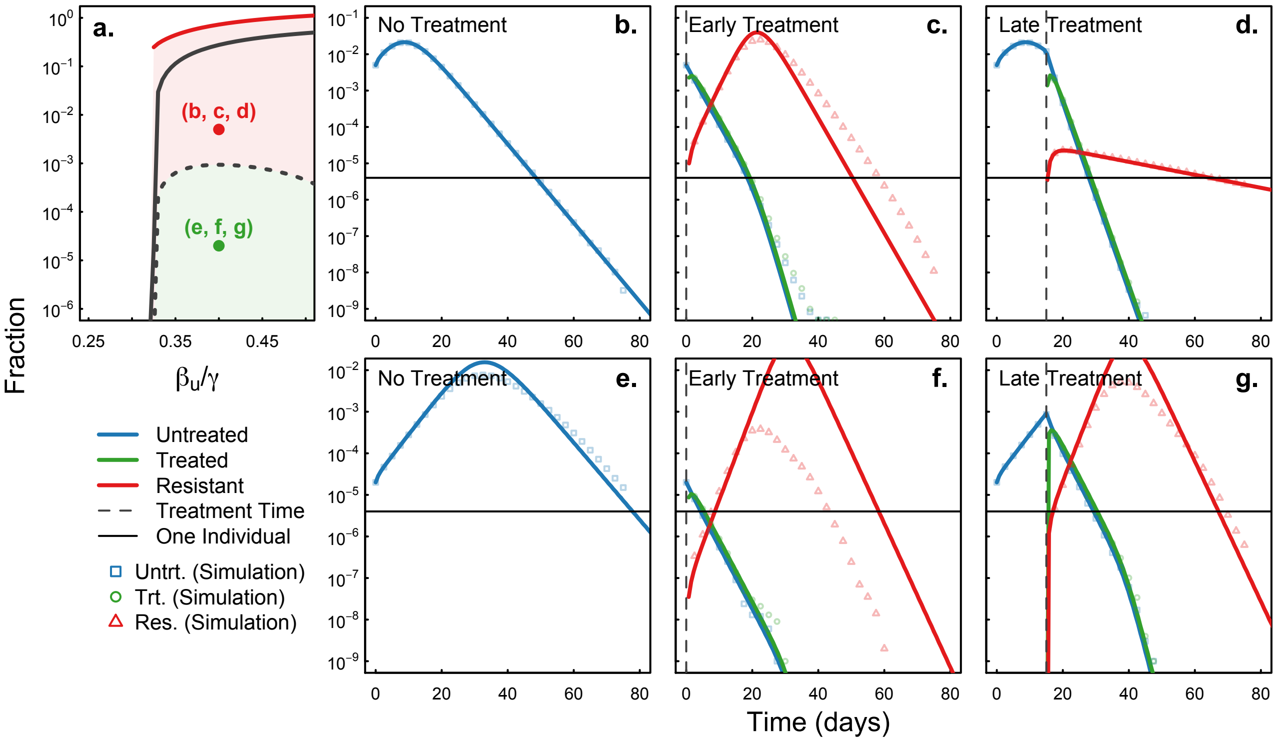

Supplemental Figure 2: **Effects of Treatment Timing on Binomial Network.** Effects of treatment when initial conditions are above (panels **b, c, d**) and below (panels **e, f, g**) the critical manifold. Panel **a** shows the critical manifold (dashed grey line) and the total number infected (red line) from the mean-field approximations, with each large dot corresponding to the panels at right. Solid lines correspond to mean-field approximations, and points correspond to means of 100,000 simulations on networks of size 250,000. Horizontal black line corresponds to a mean of 1 infected individual in a network of 250,000 over 100,000 simulations. Above the critical manifold, treatment induces large epidemics of the resistant strain (panels **c** and **d**). In the effective treatment regime (below the critical manifold) early treatment reduced the number infected by 35-fold (panel **f**) while late treatment reduces cases similarly but with much higher resistance (panel **g**). Parameters: $\beta_u = 4 \cdot 10^{-4}$, $\beta_r/\beta_u = 1.2$, $\beta_t/\beta_u = 0.3$, $\gamma_u = \gamma_t = \gamma_r = 10^{-3}$, $\rho = 0.6$, and $c = 1/500$.

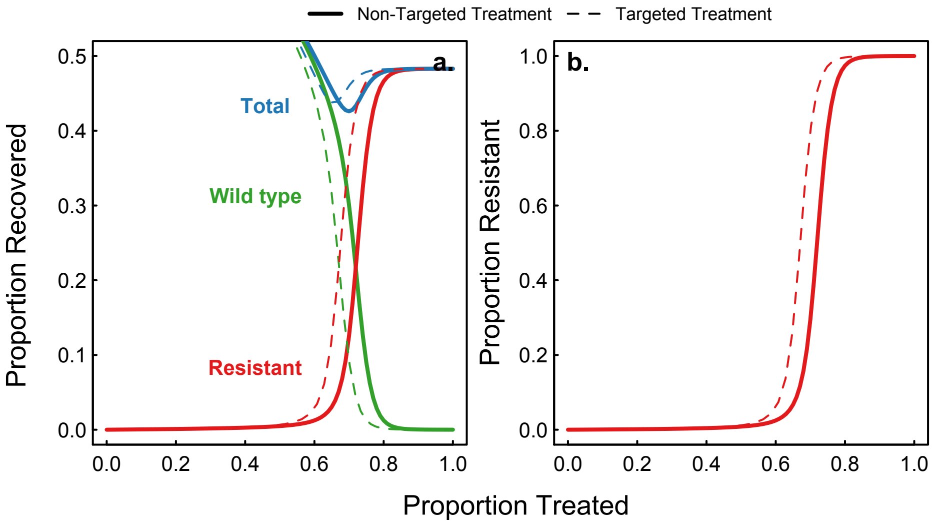

Supplemental Figure 3: **Comparison of Random and Targeted Treatment on the Binomial Network.** Panel **a** shows the final size for wild-type, resistant and both infections as a function of percentage treated, ρ, for targeted (dashed lines) and random (solid lines) treatment regimes. We see a transition from wild-type to resistant infections at a lower treatment percentage in the targeted treatment regime. Panel **b** shows the percent of total infection that is the resistant strain for the targeted (dashed line) and random (solid line) treatment. Parameters: $\beta_u = 6 \cdot 10^{-4}$, $\beta_t = 1.8 \cdot 10^{-4}$, $\beta_r = 3 \cdot 10^{-4}$, $\gamma_u = \gamma_t = \gamma_r = 10^{-3}$, and $c = 1/500$.
